# Canine infectious respiratory disease: New insights into the etiology and epidemiology of associated pathogens

**DOI:** 10.1371/journal.pone.0215817

**Published:** 2019-04-25

**Authors:** Grazieli Maboni, Mauricio Seguel, Ana Lorton, Roy Berghaus, Susan Sanchez

**Affiliations:** 1 Athens Veterinary Diagnostic Laboratory, University of Georgia, Athens, Georgia, United States of America; 2 Odum School of Ecology, University of Georgia, Athens, Georgia, United States of America; 3 Department of Population Health, College of Veterinary Medicine, University of Georgia, Athens, Georgia, United States of America; 4 Department of Infectious Diseases, College of Veterinary Medicine, University of Georgia, Athens, Georgia, United States of America; University of Lincoln, UNITED KINGDOM

## Abstract

Canine infectious respiratory disease (CIRD) is a syndrome where multiple viral and bacterial pathogens are involved sequentially or synergistically to cause illness. There is limited information regarding the prevalence of pathogens related to CIRD in the United States as well as the role of co-infections in the pathogenesis of the syndrome. We aimed to conduct a comprehensive etiologic and epidemiologic study of multiple CIRD agents in a diverse dog population using molecular methods and statistical modeling analyses. In addition, a novel probe-based multiplex real-time PCR was developed to simultaneously detect and differentiate two species of *Mycoplasma* (*M*. *canis* and *M*. *cynos*). Canine adenovirus, canine distemper virus, canine parainfluenza virus, coronavirus, influenza A virus (H3N2 and H3N8), *Bordetella bronchiseptica*, *M*. *canis*, *M*. *cynos* and *Streptococcus equi* subsp. *zooepidemicus* were investigated in specimens from clinically ill and asymptomatic dogs received at the Athens Veterinary Diagnostic Laboratory. Results showed low occurrence of classical CIRD agents such as *B*. *bronchiseptica*, canine adenovirus and distemper virus, while highlighting the potential role of emerging bacteria such as *M*. *canis* and *M*. *cynos*. Statistical modeling analyses of CIRD pathogens emphasized the impact of co-infections on the severity of clinical presentation, and showed that host factors, such as animal age, are the most important predictors of disease severity. This study provides new insights into the current understanding of the prevalence and role of co-infections with selected viruses and bacteria in the etiology of CIRD, while underscoring the importance of molecular diagnosis and vaccination against this disease.

## Introduction

Canine infectious respiratory disease (CIRD), also known as “Kennel cough”, is an endemic syndrome with multiple viral and bacterial pathogens being involved in disease causation [1]. CIRD is most common when dogs are kept in large groups with continuous intake of new animals, particularly in kennels, but also occurs in singly housed pets [2]. Clusters of infection have also been documented in veterinary hospitals [3]. Common clinical signs include nasal discharge, coughing, respiratory distress, fever, lethargy and lower respiratory tract infections [1, 3–5]. The clinical signs caused by the different pathogens associated with this syndrome are similar, which makes differential diagnosis challenging. Vaccination plays an important role in managing CIRD, and as such, several mono and multivalent vaccines are available [6]; however, despite the widespread use of vaccines to prevent CIRD, clinical disease is still common in vaccinated dogs [2, 6]. Vaccines are commercially available for some, but not all pathogens, which may explain the occasional lack of protection.

The complex multifactorial etiology of this disease involves the traditional CIRD viral and bacterial agents, canine parainfluenza virus (CPIV) [7], canine adenovirus (CAV) [8], canine distemper virus (CDV) [5], canine herpesvirus (CHV) [9], and *Bordetella bronchiseptica* [10]. New or emerging microorganisms associated with CIRD include canine influenza virus (CIV) [11], canine respiratory coronavirus (CRCov) [12], *Mycoplasma cynos* [13] and *Streptococcus equi* subsp. *zooepidemicus* (*S*. *zooepidemicus*) [14]. Other novel canine respiratory agents include canine pneumovirus [15], canine bocavirus [16], canine hepacivirus [17, 18] and canine picornavirus [19]. There is debate on whether these are truly new emerging pathogens or pre-existing pathogens that are now easier to detect due to the advent of sophisticated molecular diagnostic tools and more frequent diagnostic testing. In recent years, the role of other bacterial agents such as *Mycoplasma canis* has been questioned [13, 20]. It is unknown whether certain *Mycoplasma* species such as *M*. *canis* act as a commensal, primary or secondary agent.

The detection of co-infections of CIRD pathogens in a single dog has been previously documented [2, 12, 20]. It is most likely that a single pathogen alters the protective defense mechanisms of the respiratory tract, thereby allowing additional pathogens to infect the respiratory tissues. The presence of co-infections may increase disease severity compared with single pathogen infections [2, 5, 20]; however, the prevalence and role of co-infections in CIRD causation remain unclear.

Previous epidemiologic studies of CIRD pathogens in the United States have focused on asymptomatic dogs [21] or on specific pathogens implicated in clinical cases [11, 22, 23]; therefore, a comprehensive etiologic and epidemiologic study involving multiple CIRD agents in a diverse population of dogs has not yet been reported. Understanding disease prevalence facilitates the improvement or establishment of new vaccination programs and alternative treatments. To aid in addressing this question, we conducted a disease surveillance study using molecular methods to detect nine pathogens currently known to be involved in CIRD using samples from symptomatic and asymptomatic dogs that were received at a veterinary diagnostic laboratory. The aim was to attain information regarding pathogen occurrence according to age, seasonality, sex, clinical signs, and vaccination history. This study also aimed to evaluate the role of co-infections in disease severity, and to develop a novel probe-based multiplex real-time PCR assay to simultaneously detect and differentiate *M*. *cynos* and *M*. *canis*.

## Materials and methods

### Study population

Samples used in this study were regular submissions to the Athens Veterinary Diagnostic Laboratory (AVDL, Athens, GA, USA) by licensed veterinarians from client owned dogs for the diagnosis of respiratory disease using molecular methods. Clinical samples submitted to the AVDL are accompanied by a paper submission form, which asks questions regarding clinical history, vaccination records and clinical signs. These submission forms are then scanned and partially transcribed to an electronic database. This database was queried to retrieve all submission forms between 2011 and 2017 that requested a canine respiratory PCR panel. Signalment, clinical presentation and history of vaccination were retrieved from 559 of these electronically stored paper-based forms. The majority of these samples were received from the southeastern region of the United States. Submission forms provided no information as to whether the animals had been previously kenneled. From 2011 to 2016, the AVDL canine respiratory PCR panel included *Mycoplasma* spp., *B*. *bronchiseptica*, CAV, CDV, coronavirus (CoV) and influenza A Matrix (H3N2 and H3N8). In July 2017, PCR tests for identification of *M*. *canis*, *M*. *cynos* and *S*. *equi* subsp. *zooepidemicus* were added to the panel. Since our laboratory stores DNA for 6 months (-20°C), we were able to perform the new diagnostic tests included in the panel on all clinical samples received in 2017, by reanalyzing stored DNA. In order to investigate the presence of canine respiratory pathogens in dogs without clinical signs of respiratory disease (controls), nasal swabs were prospectively collected 4h to 24h postmortem from carcasses of asymptomatic dogs (n = 52) that were submitted to the AVDL for necropsy. These animals were selected to represent a similar age and sex distribution as the animals with clinical signs of CIRD (~ 50% males, 50% females; age mean ± SD = 2.1 ± 1.08 years). These control animals had no history of respiratory clinical disease according to the referring veterinarians and available medical records, which covered the entire life span of young animals (< 2-year-old) and at least the last year of life in older (> 2-year-old) dogs. Board-certified pathologists examined the clinical records from the asymptomatic control group, and performed complete postmortem and histological examinations to confirm that the animals were not affected by respiratory disease at the time of euthanasia. All the data collected in this study was part of routine diagnostic work-up in client-owned animals, and no additional testing or diagnostic procedures were performed for the purpose of this study. Therefore, The University of Georgia does not require Institutional Animal Care and Use Committee review and approval of such studies as long as the retrospective records review does not contain animal ID or client information. The animals investigated in this study were not identifiable in the retrospective records (e.g. only sample ID barcoding was used to identify samples).

### Nucleic acid extraction and PCR assays

Nucleic acids were extracted using QIAamp cador Pathogen Kit (Qiagen, Hilden, Germany) according to the manufacturer’s instructions using QIAcube automated nucleic acid extraction system (Qiagen). DNA samples were stored at -20°C until molecular analysis. Primers and probes are described in supplementary table 1 (S1 Table). *B*. *bronchiseptica* [24], CAV type 1 and type 2 [25], CDV [26], influenza type A [27], CoV [28], CPIV [5], and *S*. *equi* subsp. *zooepidemicus* [29] were performed as previously published. For CoV, the primers targeted the replicase gene, which is well conserved among coronaviruses, and therefore, do not allow distinction between respiratory (CRCoV) and enteric (CCoV) canine coronaviruses [28]. Further details of the PCR assays are described in supplementary table 2 (S2 Table). All assays were performed with a positive amplification control, negative amplification control, and an exogenous internal control (Qiagen Quantifast pathogen PCR and RT-PCR internal control kit). Assays were performed and results were interpreted taking into consideration the MIQE guidelines [30, 31].

### Development of a multiplex real-time PCR assay for *M*. *canis* and *M*. *cynos*

#### Primer, probe design and assay conditions

The multiplex assay was performed using CFX96 Touch system (Bio-rad, Hercules, CA, USA). Primers and TaqMan-probes (IDT DNA Technologies, Coralville, IA, USA), were manually designed using Sequencher software (Ann Arbor, MI, USA, version 5.4.6) (S1 Table). The specificity of the primers and probes was confirmed against GeneBank sequences with BLAST (http://blast.ncbi.nlm.nih.gov/Blast.cgi). Each 25μl PCR reaction mixture contained 2x real-time Quantifast mix with Rox (Qiagen), 0.8μM of each primer, 0.24μM of the TaqMan probe and 5μl of template DNA. Further details of the PCR assay are described in S2 Table. To assess clinical performance, 144 DNA samples previously tested by the *M*. *cynos* and *M*. *canis* using monoplex standard PCR assays [13] as part of the diagnostic service were analyzed using the newly developed multiplex assay and the results from both methods were compared.

#### Evaluation of PCR performance using synthetic DNA (plasmid)

For the purpose of analytical validation, the assay sensitivity and the amplification efficiency were verified by testing 10-fold serial dilutions of synthetic positive amplification controls (plasmids), using a modified approach [32]. These contained the 16S rRNA target for either *M*. *canis* or *M*. *cynos*, which were inserted into plasmids pUC57 kanamycin and transformed into competent *Escherichia coli* cells (DH5α strain). Synthetic DNA plasmids were purchased from GENEWIZ (South Plainfield, NJ, USA). Each *E*. *coli* colony-forming unit (CFU) contained one copy of the plasmid. *E*. *coli* was cultured overnight on Luria-Bertani agar with Kanamycin (Remel, San Diego, CA, USA) and isolated colonies were used to prepare a 10-fold serial dilution from a starting 0.5 McFarland concentration. Each dilution was plated on McConkey agar (Remel) in triplicate (100μl). Colony counting was performed after 24h of incubation at 37°C. DNA was extracted from each dilution as described above. The limit of detection (LOD) was defined as the lowest concentration at which 95% of the positive samples were detected [30]. The concentration (plasmid copy number) in each dilution was calculated from the plate counts (CFU/mL). The copy number in each PCR reaction was calculated based on the extraction volume (200μl), the final elution volume (120μl) and the volume per reaction (5μl); assuming error free conditions. Dilutions were repeated in triplicate runs for each targeted microorganism and standard curves were constructed from the Cq values. PCR efficiency was calculated according to MIQE guidelines [30, 31].

#### Inclusivity and exclusivity testing

*M*. *canis* (ATCC 19525), *M*. *cynos* (ATCC 27544) and synthetic DNA (plasmids) from both targets were used to test the inclusivity of the assay. Assay exclusivity included 7 different species of *Mycoplasma* spp. and 12 strains of bacteria and viruses associated with respiratory diseases, including DNA extracted from Nobivac vaccine (Merck, Kenilworth, NJ, USA) (Table 1). Genus and species identification of strains isolated at AVDL were confirmed by Sanger sequencing as previously described [33].

**Table 1:** Assessment of the specificity of the multiplex TaqMan real-time PCR assay for *M*. *canis* and *M*. *cynos* by testing different viral and bacterial strains.

Microorganism	Strain/source	*M*. *canis* PCR	*M*. *cynos* PCR
***Mycoplasma* spp**.			
*Mycoplasma canis*	ATCC 19525	+	-
*Mycoplasma cynos*	ATCC 27544	-	+
*Mycoplasma canis*	Synthetic DNA	+	-
*Mycoplasma cynos*	Synthetic DNA	-	+
*Mycoplasma bovis*	ATCC 2738	-	-
*Mycoplasma cavipharyngis*^#^	AVDL	-	-
*Mycoplasma insons*^#^	AVDL	-	-
*Mycoplasma haemofelis*	AVDL	-	-
*Mycoplasma haemonitum*	AVDL	-	-
*Mycoplasma haemalamae*	AVDL	-	-
*Mycoplasma pulmonis*	ATCC 19612	-	-
***Bacteria and viruses associated with respiratory diseases***			
*Ureaplasma urealyticum*	ATCC 27618	-	-
Bordetella bronchiseptica*	AVDL	-	-
Bovine respiratory syncytial virus	AVDL	-	-
Canine parainfluenza virus*	Synthetic DNA	-	-
Canine respiratory coronavirus*	Synthetic DNA	-	-
Influenza type A*	AI (USDA-203ADV0704)	-	-
*Mannheimia haemolytica*	AVDL A11-05837	-	-
Nobivac vaccine	Merck	-	-
Pan-paramyxovirus	AVDL	-	-
*Pasteurella multocida*	AVDL	-	-
*Pasteurella pneumotropica*	AVDL	-	-
*Streptococcus equi sub*. *zooepidemicus*	ATCC 9528	-	-

*Pathogens tested in the canine respiratory panel offered by AVDL.

+ Positive multiplex real-time PCR assay.–Negative multiplex real-time PCR assay.

All microorganisms were tested in duplicates.

^#^Microorganisms were obtained from AVDL clinical cases and were previously sequenced to confirm identity.

### Statistical and data analyses

The signalment (age, sex), dates (seasonality), reported clinical signs and vaccination status were recorded for each animal from the sample submission form received at AVDL. To assess the effect of season on the occurrence of CIRD pathogens, seasonality was divided into cold and warm seasons, which are the two clear predominant seasons in the state of Georgia, United States. The cold season was defined as October 15^th^ to April 15^th^, when temperatures vary from 1°C to 22°C. The warm season was defined as April 16^th^ to October 14^th^, when temperatures vary from 11°C to 31°C. The rate of pathogen detection by age was evaluated in four categories defined as puppyhood (1 to 12 month-old), adolescence (1–3 year-old), adulthood (4–8 year-old) and senior (>8-year-old). Clinical signs were separated in the three following categories: score 0 was given to asymptomatic dogs sampled at necropsy (absence of clinical, macroscopic and histologic signs of respiratory diseases), score 1 was given to dogs with mild clinical signs, and score 2 was given to animals presenting with moderate to severe clinical signs (Table 2). Differences in the rate of detection of CIRD pathogens across life stages, seasons, clinical sign categories and sex were assessed by Fisher’s Exact test using Stata statistical software (StataCorp LLC, College Station, TX, USA, version 15.1). Pairwise comparisons between categories were performed using the Bonferroni procedure to limit the overall type I error rate to 5%. Confidence intervals for the rate of detection were calculated using the exact method in Graphpad software (http://graphpad.com/quickcalcs/confInterval2/). P-values≤ 0.05 were considered significant.

**Table 2:** Clinical scores of respiratory signs from dogs at the time of sample collection.

Clinical score	Clinical signs	Total number of dogs
0 (asymptomatic)	No history of respiratory disease	n = 52
1 (mild)	Cough or sneeze or nasal discharge	n = 213
2 (moderate/severe)	Cough or sneeze or nasal discharge, in addition to one of the following signs:Fever or lethargy/depression or inappetance or pneumonia	n = 223

Number of dogs presenting the following clinical signs at the time of sample collection: cough n = 311; sneeze n = 160; nasal discharge n = 10; fever n = 123; lethargy/depression n = 46; inappetance n = 94; pneumonia n = 22. Clinical signs history was not described in 129 forms.

In order to determine whether the presence of more than one pathogen (co-infection) was associated with the severity of respiratory disease (mild or severe clinical scores), the cases were separated into groups of dogs that were infected with one (single infection) or more than one pathogen (co-infected). The effect of the presence or absence of co-infections was assessed through Fisher’s Exact test using clinical sign scores (mild vs severe) as the categorical outcome. In order to determine whether specific pathogens were more likely to be associated with severe clinical disease, a 3D network analysis was performed [34, 35]. Based on the results of these analyses, the associations between clinical scores and CPIV+*M*. *canis*, CPIV+*M*. *cynos*, CPIV+*M*. *cynos+M*. *canis*, CPIV+*B*. *bronchiseptica*, *B*. *bronchiseptica*+*M*. *cynos*+CPIV, CPIV+*M*. *cynos*+*M*. *canis* infections were assessed using binomial generalized linear models. Potential predictors included sex, age, the total number of infections, and the presence of *M*. *cynos*, *M*. *canis*, *B*. *bronchiseptica* and CPIV single infections with clinical score (mild vs severe) as a binomial response. Predictors were added and excluded based on the analysis of each model output (Akaike’s Information Criteria, P values, coefficients, and deviance), and all fitted models were ranked based on second order Akaike’s Information Criteria (AICc) using the “MuMln” package in R statistical software [36] (R core development team, Vienna, Austria, version 3.2.2). Statistical inference was performed based on outputs of top ranked models (delta AICc <2.0). These models were averaged and final outputs reported for explanatory purposes [35].

## Results

### Development of a multiplex real-time PCR for detection of *M*. *canis* and *M*. *cynos* from clinical specimens

#### LOD and linearity of standard curves using synthesized DNA

Standard curves were constructed using mean Cq values from triplicate 10-fold dilutions of *M*. *canis* and *M*. *cynos* synthetic DNA. *M*. *canis* standard curve assay had a slope of -3.430 (R^2^ 0.999) corresponding to an amplification efficiency of 95.7%. *M*. *cynos* standard curve had a slope of -3.421 (R^2^ 0.999) corresponding to an amplification efficiency of 96.03%. For *M*. *canis* the LOD was equivalent to 2.6 CFU/reaction, equivalent to approximately 2.6 copies of *M*. *canis* genome per reaction. For *M*. *cynos* the LOD was 3.08 CFU/reaction, equivalent to 3.08 copies of *M*. *cynos* genome per PCR reaction.

#### Inclusivity and exclusivity testing

The developed assay showed 100% inclusivity for the two *M*. *canis* and the two *M*. *cynos* samples tested (ATCCs and synthetic DNAs), and 100% exclusivity for seven non-target viral DNA and 13 non-target bacterial DNA, including different species of *Mycoplasma* (Table 1).

#### Clinical performance using *M*. *canis* and *M*. *cynos* primer/probes on previously stored DNA

Monoplex and multiplex assays presented different results for 5/144 samples targeting *M*. *canis* and 5/144 samples when targeting *M*. *cynos*. Thus, the comparison of these two PCR systems showed an individual agreement of 96.53% for each *M*. *canis* and *M*. *cynos*.

### Detection of CIRD agents by season, sex, age, clinical condition and vaccination status

**Fig 1:**
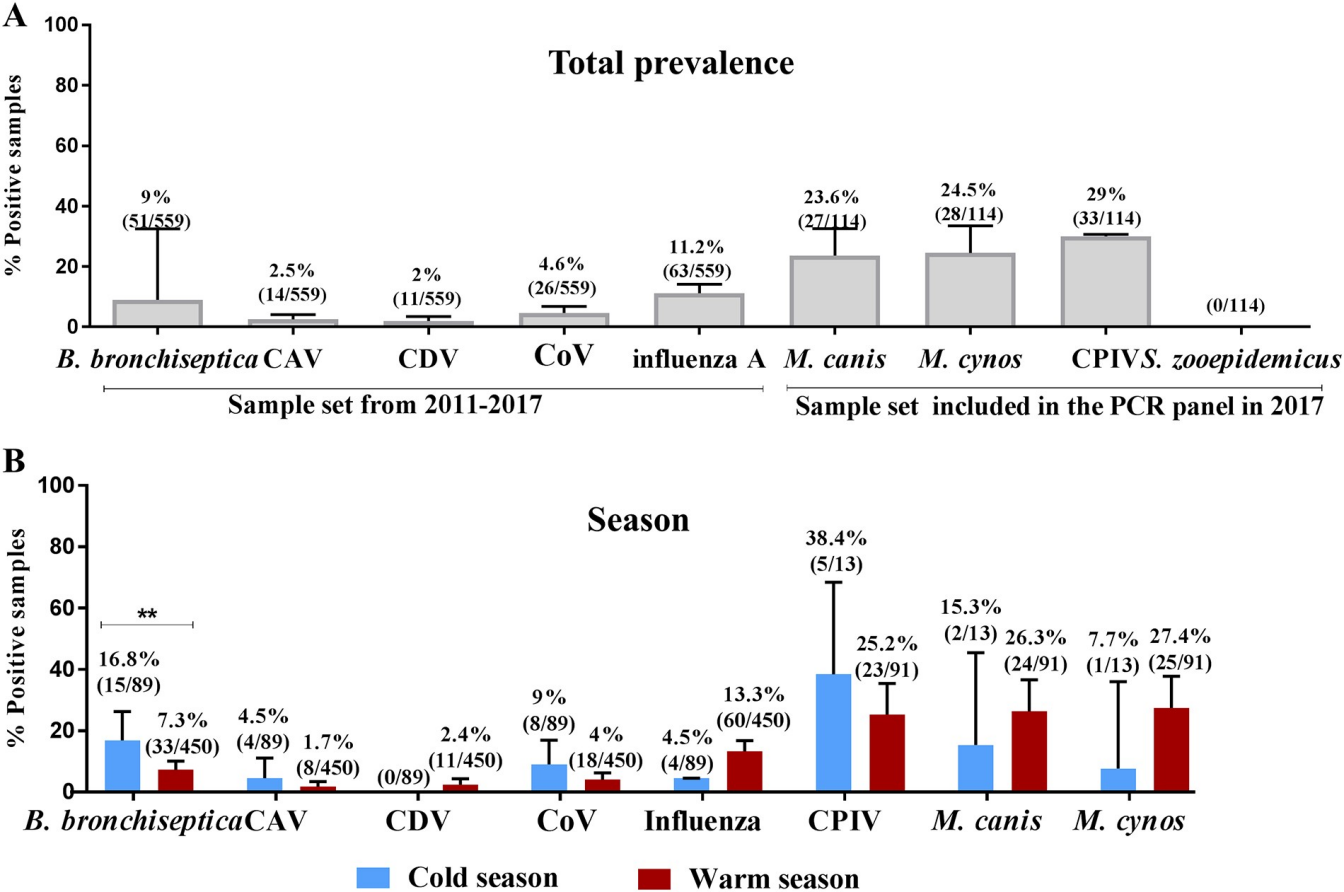
Rate of detection of canine infectious respiratory disease pathogens detected by PCR assays in a veterinary diagnostic laboratory. **(A)** Total detection of pathogens in respiratory specimens from 2011 to 2017. **(B)** Rate of detection according to season. Two seasons, cold and warm, were determined based on the average temperatures of the state of Georgia (USA). *Streptococcus equi* subsp. *zooepidemicus* was not displayed in graphs B, C and D because all samples were negative for this pathogen. Error bars represent 95% confidence intervals. Data were analyzed using Fisher`s Exact test: * P < 0.05, **P < 0.01, ***P < 0.001.

**Fig 2:**
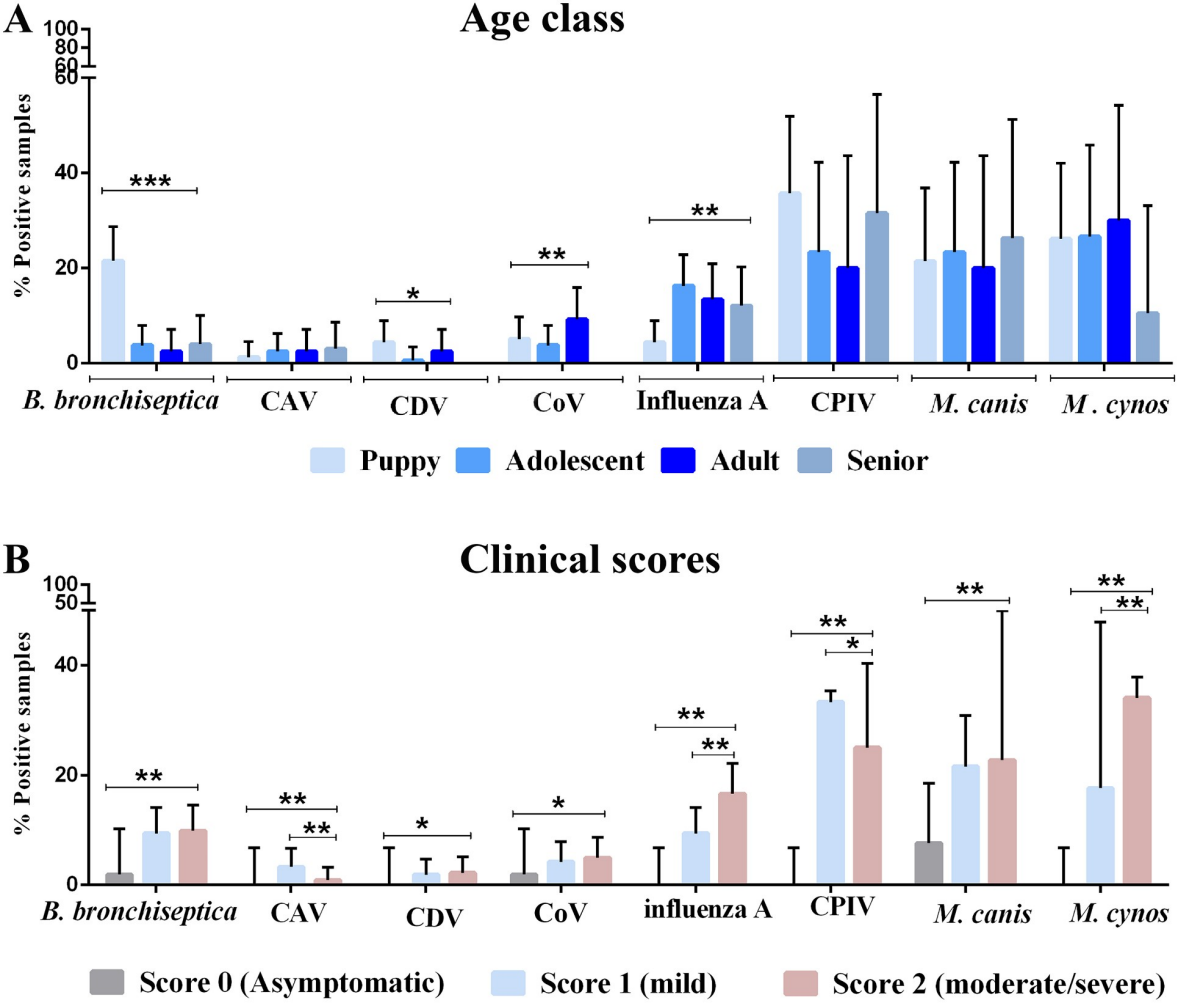
Rate of detection of canine infectious respiratory pathogens by age (A) and clinical presentation (scores) (B). Age class: puppyhood (0 to 12 months, n = 158), adolescence (1 to 3 years, n = 160), adulthood (4 to 8 years, n = 119) and seniors (≥ 8 years, n = 99). Sample set included in the PCR panel in 2017 (canine parainfluenza virus, *Mycoplasma canis* and *M*. *cynos*): puppyhood n = 42, adolescent n = 30, adulthood n = 20, senior n = 19 (A). Clinical score 0 (asymptomatic dogs, n = 52), score 1 (cough or sneeze or nasal discharge, n = 213), score 2 (signs from score 1 in addition to fever or lethargy/depression or inappetence or pneumonia, n = 223) (B). All samples were negative for *Streptococcus equi* subsp. *zooepidemicus*. Error bars represent 95% confidence intervals. Data were analyzed using Fisher`s Exact test and Bonferroni correction: * P < 0.05, **P < 0.01, ***P < 0.001.

Results of molecular detection of pathogens associated with CIRD are illustrated in Figs 1 and 2. CPIV (29%, 33/114), *M*. *canis* (23.6%, 27/144) and *M*. *cynos* (24.5%, 28/144) were the most commonly detected pathogens followed by influenza A (11.2%, 63/559), *B*. *bronchiseptica* (9%, 51/559), CoV (4.6%, 26/559), CAV (2.5%, 14/559) and CDV (2%, 11/559). All samples were negative for *S*. *equi* subsp. *zooepidemicus* (Fig 1A). AVDL received more samples to be tested by the canine respiratory panel during warm months (n = 450) than during cold months (n = 89). There was no significant difference in the rate of detection of pathogens, except for *B*. *bronchiseptica*, which was more prevalent during the cold season (16.85%, 15/89) compared to the warm season (7.33%, 33/450) (P ≤ 0.001) (Fig 1B). Likewise, there was no significant association of CIRD pathogens with sex, except for *B*. *bronchiseptica*, which was more commonly detected in female (12.4%, 30/241) than in male dogs (5.2%, 15/288) (P ≤ 0.01). CIRD pathogens were present in animals of all age categories; however, puppies were more commonly infected with *B*. *bronchiseptica* and CDV compared to other age groups (P ≤ 0.01 and P ≤ 0.05, respectively). CoV was more prevalent in adults (9.24%, 11/119) compared to other age classes (P ≤ 0.001). Influenza A virus was less common in puppies (4.43%, 7/158) than in older age categories (P ≤ 0.001) (Fig 2A). All assessed pathogens were found more frequently in animals with clinical signs (mild or severe) compared to asymptomatic dogs (Fig 2B). This included *M*. *canis*, commonly considered a commensal in the canine respiratory tract, which was more frequently identified in symptomatic dogs than in asymptomatic dogs (P ≤ 0.01). Asymptomatic dogs were only positive for *B*. *bronchiseptica* (n = 1/52), CoV (n = 1/52) and *M*. *canis* (n = 4/52).

Vaccination status was described in 152/559 forms submitted to AVDL. These forms did not contain information regarding when animals were vaccinated. According to the information retrieved from these forms, dogs vaccinated against some of the CIRD pathogens presented with mild to severe clinical signs of respiratory disease and were PCR positive for *B*. *bronchiseptica* (n = 9), CDV (n = 4), CPIV (n = 3) and influenza A (n = 1).

### Effect of co-infections on clinical signs

**Fig 3:**
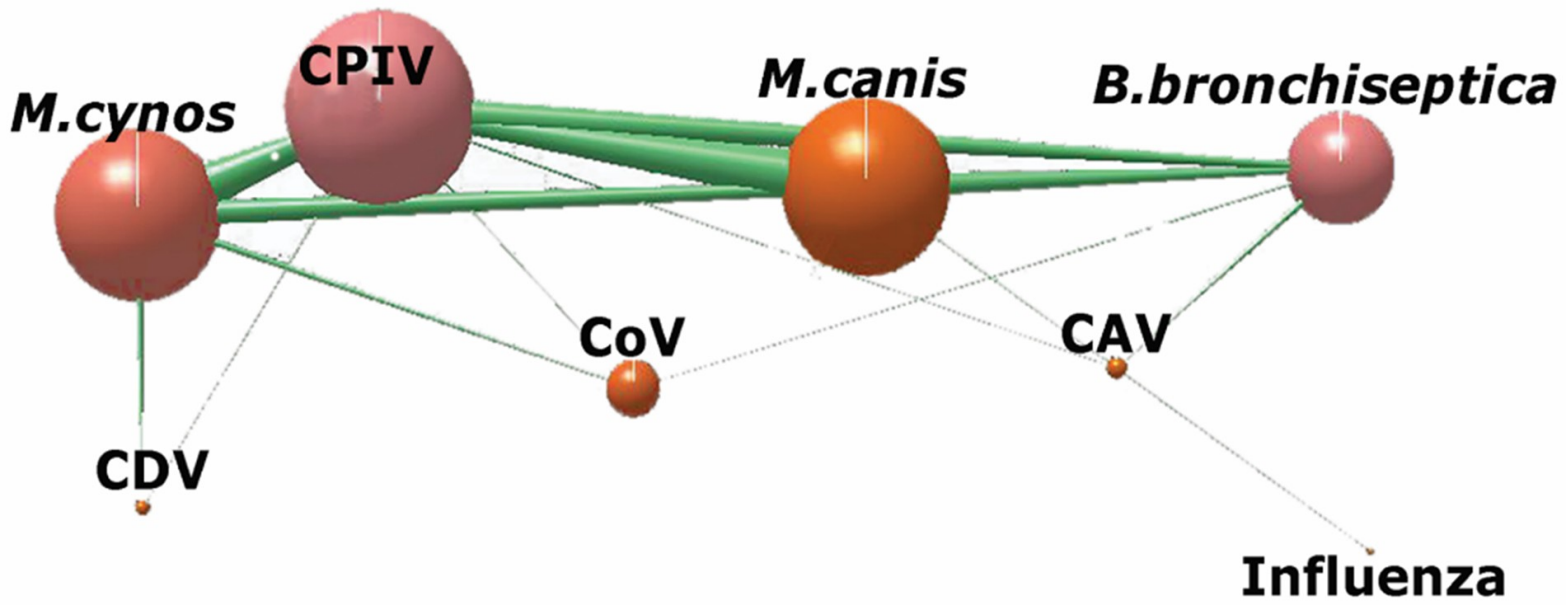
Visualization of CIRD co-infections interactions in Network 3D, n = 95 (Williams, 2010; Yoon et al., 2004). The network shows strong connectivity between CPIV+*M*. *canis*, CPIV+*M*. *cynos*, CPIV+*M*. *cynos+M*. *canis*, CPIV+*B*. *bronchiseptica*, *B*. *bronchiseptica*+*M*. *cynos*+CPIV, CPIV+*M*. *cynos*+*M*. *canis*.

Dogs with co-infections presented with severe clinical signs more often than dogs with single infections (CI = 0.26–0.63, P < 0.0001). Based on the network analysis (n = 95), the most common and strongest co-infection associations were *M*. *cynos+*CPIV, *M*. *canis+*CPIV, *M*. *cynos*+CPIV+*M*. *canis*, CPIV+*B*. *bronchiseptica* and *M*. *canis*+*B*. *bronchiseptica* (Fig 3). Based on the results of the multimodel multivariate analyses, young age was the most significant predictor of severe clinical signs (age estimate (years) = -0.5 ± 0.21, Z = 2.32, P = 0.02) (Table 3). Although the presence of *M*. *cynos* or CPIV and the association between these two pathogens (co-infection) was retained in all top ranked models, their effect on the severity of clinical signs was not significant (Table 3, S3 Table).

**Table 3:** Multimodel coefficients estimate, standard error (SE), Z and P values for the averaged top binomial generalized linear models with clinical score (mild or severe) as response.

Predictor	Estimate	SE	95% Confidence Interval	Relative Prevalence	Z	P value
Age	-0.5	0.21	-0.92, 0.078	0.60	2.32	0.02
*M*. *cynos*	0.33	0.49	-0.27, 1.71	2.05	0.67	0.5
CPIV	-0.13	0.34	-1.61, 0.34	0.52	0.384	0.7
Sex (male)	0.05	0.2	-0.47, 1.23	1.46	0.247	0.8
CPIV-*M*.*cynos*	0.1	0.42	-0.98, 2.54	2.17	0.24	0.8
Number of infections	0.14	0.24	-0.33, 0.63	1.15	0.59	0.55

## Discussion

This study provided new insights into the etiology and epidemiology of CIRD associated pathogens using a molecular surveillance approach in a veterinary diagnostic laboratory. We explored two main aspects: (i) the rate of detection of nine CIRD associated pathogens by age, season, sex, clinical signs, and vaccination status; and (ii) the effect of co-infections on the severity of clinical disease. Our results indicated that the presence of co-infections and young age were associated with the severity of clinical signs. Additionally, we found a low occurrence of classical CIRD pathogens such as *B*. *bronchiseptica*, CAV and CDV, while identifying a higher than expected detection of bacterial agents such as *M*. *canis* and *M*. *cynos*.

The low detection rate of traditional CIRD agents such as *B*. *bronchiseptica*, CAV and CDV might be associated with the extensive vaccination programs adopted in the United States, which may have reduced the circulation of these pathogens in the canine population. In fact, our data show that very few clinically ill animals (n = 17), previously vaccinated against *B*. *bronchiseptica*, CDV, CPIV and influenza, were PCR positive for these agents. This result emphasizes the important role of CIRD vaccination regimes and suggests that currently available vaccines against *B*. *bronchiseptica*, CAV and CDV are effective. Similar findings were reported in a large surveillance study across Europe, where disease occurrence was significantly reduced in dogs vaccinated against classical CIRD pathogens (CDV, CAV-2 and CPIV) [2].

It is noteworthy that CPIV, traditionally considered one of the most important viral agents of CIRD, was the most commonly detected microorganism in our study (29%, 33/114), and it was significantly associated with mild and moderate/severe clinical signs. Previous investigations on canine respiratory viruses have found high prevalence of CPIV in clinically ill dogs [5, 20, 37], and a Canadian study reported CPIV as the cause of a respiratory disease outbreak in a veterinary hospital [3]. Influenza is a newly described CIRD respiratory virus [11, 12]. In the United States, canine influenza is caused by two subtypes: H3N8 and H3N2. Canine H3N8 was first identified in 2004 in racing greyhounds in Florida, United States [11], and it has been reported that this strain developed from an equine H3N8 influenza strain that was transmitted from horses to dogs [11]. Canine H3N2 was first identified in the United States in March 2015, following an outbreak of respiratory illness in dogs from Chicago [38]. Between 2011 and 2017, we found 57 PCR positive samples for influenza A (including H3N8 and H3N2); interestingly, 51/57 of these positive samples were processed in 2015, which coincides with the H3N2 canine influenza outbreak in the United States. To date, there is no evidence of spread of canine influenza viruses from dogs to people [39]; however, special attention is warranted due to the risk of viral genetic reassortment. Therefore, continued monitoring of canine influenza by veterinary and human health agencies is of critical importance.

*M*. *cynos* is an emerging bacteria implicated in canine respiratory disease [40], which was shown to cause pneumonia in dogs after experimental endobronchial inoculation [4]. Despite the clear involvement of *M*. *cynos* in lower respiratory tract infections [4, 13, 41], there is limited information on the prevalence and pathogenesis of this agent. In this study, *M*. *cynos* was one of the most prevalent pathogens (24.5%), and it was more commonly identified in symptomatic than asymptomatic dogs. In the first group, there was a significant association between the presence of this pathogen and the development of moderate and severe clinical signs. Similar findings were observed in a large rehoming kennel where *M*. *cynos* was most common in dogs with moderate signs of CIRD [13]. The role of other *Mycoplasma* species in canine respiratory infections, including *M*. *canis*, has been a recent topic of research and debate. In this study, we found a significant association between the presence of *M*. *canis* and the severity of clinical signs. *M*. *canis* is considered part of the microbiota of the upper respiratory tract of dogs [13, 42], and was previously found not to be significantly associated with respiratory disease [13]. Our interest in this microorganism was triggered by a recent study where *M*. *canis* was associated with acute respiratory disease in dogs [20], which along with our findings provide further insights into a potential role of *M*. *canis* in CIRD. In addition, *M*. *canis* appears to be a common co-infection and warrants further consideration by veterinarians and diagnostic laboratories.

Dogs with viral or bacterial co-infections present with moderate to severe clinical signs more often than dogs with single infection. The prevalence of these co-infections have been previously reported [2, 20, 37, 43]; however, to the best of our knowledge, none of the prior investigations have performed a detailed assessment on the effect of co-infections in the severity of clinical disease. The 3D network analysis showed that *M*. *cynos*, CPIV, *M*. *canis* and *B*. *bronchiseptica* were the most common pathogens seen in co-infections, with *M*. *cynos* and CPIV being the most frequent pathogen combination. Knowledge regarding the most common co-infections may help clinicians determine appropriate treatment strategies, improve patient outcomes, and facilitate antimicrobial stewardship.

In this study, the age of the animals was the most significant predictor of moderate to severe clinical signs. Other studies have reached similar conclusions [2, 13, 44], indicating that host factors, such as age, are important in CIRD severity. Young dogs may have lower levels of immunity against pathogens associated with CIRD, and may be subjected to crowded conditions more often, which could increase their susceptibility to infection and lead to more severe clinical signs.

It was challenging to find an ideal control group for a retrospective and diagnostic-based surveillance study. Our approach was to include necropsied dogs in order to ensure the absence of lesions in the respiratory tract. We carefully selected the necropsied dogs based on age, clinical history, macroscopic and histologic findings. The likelihood of detecting pathogens in post-mortem samples may differ; however, based on prior investigations on viral and bacterial persistence on inanimate surfaces and cadavers, we believe that the integrity of the nucleic acid material from the microorganisms was maintained. Our assumption is supported by other studies showing that *Bordetella pertussis* persisted on inanimate surfaces for 3–5 days, influenza virus for 1–2 days and adenovirus for 7 days [40] [41] [42,43]. Tubercle bacilli was isolated from cadavers within 24 hours post-mortem [44], and viable human immunodeficiency virus was recovered from patients at autopsy 6 to 16 days after death [45, 46]. Furthermore, the pathogens surveyed in our study are routinely detected from nasal swabs collected during necropsy from dogs diagnosed with respiratory disease in our diagnostic laboratory.

This study has some limitations. First, the PCR-based assays could potentially yield false-positive results in dogs that recently received modified live vaccines containing the specific pathogens of interest. This assumption is based on findings from a previous study showing that PCR assays may detect vaccine content within 28 days after vaccination with modified live vaccines against CAV, *B*. *bronchiseptica* and CPIV [45]. Since the time between vaccination and the onset of clinical CIRD was not documented in our submission forms, we cannot say with certainty whether positive PCR results corresponded to field or vaccine strains. Secondly, as with any pathogen detection test, the PCR assays may yield false-negative results if samples are collected at a point in the disease process when pathogens are not present at detectable levels [46].

## Conclusion

This study provided new insights into the current understanding of the rate of detection of CIRD pathogens in the United States and the role of co-infections in disease severity, highlighting the importance of PCR panels for fast diagnostics. Key findings were that younger dogs and those with a higher number of co-infections are more likely to develop severe clinical signs, underscoring the importance of vaccination against CIRD at an early age. Our findings also highlight the low occurrence of classical CIRD agents such as *B*. *bronchiseptica*, CAV and CDV, while emphasizing the potential role of emerging bacteria such as *M*. *canis* and *M*. *cynos*. The developed real-time PCR assay for simultaneous detection of *M*. *cynos* and *M*. *canis* in clinical specimens provided results within 2h in a highly standardized format, representing a fast and efficient diagnosis alternative. The information presented here will help veterinarians obtain a timely etiologic diagnosis, and facilitate the selection of appropriate therapies and disease control measures.

## Supporting information

S1 Table Primers and probes used to detect pathogens associated with canine infectious respiratory diseases. F: forward primer; R: reverse primer; P: probe.
(DOCX)

S2 Table Standard and real-time PCR conditions used to detect pathogens associated with canine infectious respiratory diseases. CAV: canine adenovirus; CDV: canine distemper virus; CCov: canine respiratory coronavirus; CPIV: canine parainfluenza virus. Reagent kits (Qiagen, Hilden, Germany). *Canine vaccine Nobivac 1-DAPPV (Merck, Kenilworth, NJ, USA). # Plasmids pUC57-kanamycin (Genewiz, South Plainfield, NJ, USA). Standard T100 thermo-cycler and Mycycler system (Bio-rad, Hercules, CA, USA). Applied Biosystem 7500 thermo-cycler (Thermofisher, Waltham, MA, USA).
(DOCX)

S3 Table Top ranked binomial generalized linear models for severity of clinical signs (mild vs severe) as response in dogs with CIRD. Models are ranked based on second order Akaike’s Information Criteria (AICc). * Sample size for all models was n = 95, which corresponded to the subset of animals studied with complete data on co-infections and molecular diagnostics. *M*. *cynos* = *Mycoplasma cynos*, CPIV = canine parainfluenza virus, *M*. *canis* = *Mycoplasma canis*, *B*. *bronchiseptica* = *Bordetella bronchiseptica*.
(DOCX)
