# Supplementary material for: Canine infectious respiratory disease: New insights into the etiology and epidemiology of associated pathogens
Source: PLoS One. 2019 Apr 25;14(4):e0215817. doi: 10.1371/journal.pone.0215817 (PMC6483346; doi:10.1371/journal.pone.0215817)
Supplement: S1 Table — F: forward primer; R: reverse primer; P: probe. (DOCX) [file pone.0215817.s001.docx]

| **Target** | **Gene** | **Primer/Probe** | **Sequence 5`-3`** | **Reference** |
| --- | --- | --- | --- | --- |
| *B. bronchiseptica* | *Flagellin* | F | AGGCTCCCAAGAGAGAAAGGCTT | Hozbor et al 1999 |
|  |  | R | TGGCGCCTGCCCTATC |  |
| CDV | *Nucleoprotein* | F | ACTATTGAGAGACCTCCAGCTGAAA | Modified from Saito et al 2006 |
|  |  | R | TGCGGTATCCTTCGGTTTGT |  |
|  |  | P | CCGATTGCCGAGCTAGACTCTTTGTCA  /56-FAM/3BHQ-1/ |  |
| Cov | *Replicase* | F | TGATGATGSNGTTGTNTGYTAYAA | Escutanaire et al 2007 |
|  |  | R | GCATWGTRTGYTGNGARCARAATT C |  |
| CAV | *E3* | F  R | CGCGCTGAACATTACTACCTTGTC  CCTAGAGCACTTCGTGTCCGCTT | Chaturvedi et al 2008 |
|  |  |  |  |  |
| Influenza | *Matrix protein 2* | F | AGATGAGTCTTCTAACCGAGGTCG | Spackman et al 2003 |
|  |  | R | TGCAAAAACATCTTCAAGTCTCTG |  |
|  |  | P | TCAGGCCCCCTCAAAGCCGA  /56FAM/ZEN/3IABkFQ/ |  |
| CPIV | *Nucleocapsid protein* | F (PNP1) | AGTTTGGGCAATTTTTCGTCC | Erles et al 2004 |
|  |  | R (PNP2 | TGCAGGAGATATCTCGGGTTG |  |
|  |  | F (PNP3) | CGTGGAGAGATCAATGCCTATGC |  |
|  |  | R (PNP4) | GCAGTCATGCACTGCAAGTCACTA |  |
| *S. zooepidemicus* | *sodA* | F | AGAGCAATTCACAGCAGCA | Baverud et al 2007 |
|  |  | R | ACCAGCCTTATTCACAACCA |  |
|  |  | R | ACCGGCTTGGTTAACCACTA |  |
|  |  | P | CAGGCCCAACCTGAGCCAAA |  |
| *M. canis* (uniplex) | *16S rRNA* | F (general) | CACCGCCCGTCACACCA | Chalker et al 2004 |
|  |  | R | CTGTCGGGGTTATCTCGAC |  |
| *S. zooepidemicus* | *sodA* | F | AGAGCAATTCACAGCAGCA | Baverud et al 2007 |
|  |  | R | ACCAGCCTTATTCACAACCA |  |
|  |  | R | ACCGGCTTGGTTAACCACTA |  |
|  |  | P | CAGGCCCAACCTGAGCCAAA |  |
| *M. cynos* (uniplex) | *16S rRNA* | R | GATACATAAACACAACATTATAATATTG | Chalker et al 2004 |
| *M. canis+M. cynos* (multiplex) | *16S rRNA* | F (general)  R (general)  Probe *M. canis*  Probe *M. cynos* | TTAACTACGGAGACAACTG  TGAAAACTGAATAGTAAAGATA  TTATCAATTATTATTTTAAATGTCA  TATAATGTTGTGTTTATGTATC | This study |

**S1 Table. Primers and probes used to detect pathogens associated with canine infectious respiratory diseases.**

F: forward primer; R: reverse primer; P: probe
