# Supplementary material for: Canine infectious respiratory disease: New insights into the etiology and epidemiology of associated pathogens
Source: PLoS One. 2019 Apr 25;14(4):e0215817. doi: 10.1371/journal.pone.0215817 (PMC6483346; doi:10.1371/journal.pone.0215817)
Supplement: S2 Table — CAV: canine adenovirus; CDV: canine distemper virus; CCov: canine respiratory coronavirus; CPIV: canine parainfluenza virus. Reagent kits (Qiagen, Hilden, Germany). *Canine vaccine Nobivac 1-DAPPV (Merck, Kenilworth, NJ, USA). # Plasmids pUC57-kanamycin (Genewiz, South Plainfield, NJ, USA). Standard T100 thermo-cycler and Mycycler system (Bio-rad, Hercules, CA, USA). Applied Biosystem 7500 thermo-cycler (Thermofisher, Waltham, MA, USA). (DOCX) [file pone.0215817.s002.docx]

| **Target** | **PCR format/**  **Thermo-cycler** | **Amplification conditions** | **Positive control** |
| --- | --- | --- | --- |
| *B. bronchiseptica* | Standard (T100) | 50°C – 30 min  95°C – 15 min  40 cycles: 95°C – 15 sec  50°C – 20 sec  72°C – 30 sec | AVDL *B. bronchiseptica* sequenced strain |
| CAV | Standard (T100) | Same as *B. bronchiseptica* | Nobivac 1* |
| CDV | Standard (T100) | 50°C – 30 min  95°C – 15 min  40 cycles: 95°C – 15 sec  50°C – 30 sec  72°C – 30 sec | Nobivac 1* |
| Cov | Standard (MyCycler) | Same as CDV | Plasmids pUC57-kanamycin *ORF1b* gene # |
| Influenza A | Real-time (Smart Cycler) | Same as CDV | Influenza type A (USDA catalog  number 203ADV0704) |
| CPIV | Standard (T100) | Same as CDV | Plasmid pUC57-kanamycin *16S rRNA* gene # |
| *S. zooepidemicus* | Real-time (Smart Cycler) | 95°C – 5 min  40 cycles: 95°C – 5 sec  50°C – 30 sec | *S.* *zooepidemicus* ATCC 700400 |
| *M. canis* (uniplex) | Real-time (AB 7500) | 95°C – 5 min  40 cycles: 95°C – 5 sec  55°C – 30 sec  68°C – 30 sec | Plasmid pUC57-kanamycin *16S rRNA* gene # |
| *M. cynos* (uniplex) | Real-time (AB 7500) | Same as *M. canis* | Plasmid pUC57-kanamycin *16S rRNA* gene # |
| *M. canis* + *M. cynos* (multiplex) | Real-time CFX96 Touch system | 95°C – 10 min  45 cycles: 95°C – 30 sec  60°C – 1 min | Plasmid pUC57-kanamycin *16S rRNA* gene # |

**S2 Table. Standard and real-time PCR conditions used to detect pathogens associated with canine infectious respiratory diseases.**

CAV: canine adenovirus; CDV: canine distemper virus; Cov: coronavirus; CPIV: canine parainfluenza virus; *S. zooepidemicus*: *Streptococcus equi* subspecies *zooepidemicus*

Reagent kits (Qiagen, Hilden, Germany)

*Canine vaccine Nobivac 1-DAPPV (Merck, Kenilworth, NJ, USA)

# Plasmids pUC57-kanamycin (Genewiz, South Plainfield, NJ, USA)

Standard T100 thermo-cycler and Mycycler system (Bio-rad, Hercules, CA, USA)

Applied Biosystem 7500 thermo-cycler (Thermofisher, Waltham, MA, USA)
