# Supplementary material for: Canine infectious respiratory disease: New insights into the etiology and epidemiology of associated pathogens
Source: PLoS One. 2019 Apr 25;14(4):e0215817. doi: 10.1371/journal.pone.0215817 (PMC6483346; doi:10.1371/journal.pone.0215817)
Supplement: S3 Table — * Sample size for all models was n = 95, which corresponded to the subset of animals studied with complete data on co-infections and molecular diagnostics. M. cynos = Mycoplasma cynos, CPIV = canine parainfluenza virus, M. canis = Mycoplasma canis, B. bronchiseptica = Bordetella bronchiseptica. (DOCX) [file pone.0215817.s003.docx]

**S3 Table. Top ranked binomial generalized linear models for severity of clinical signs (mild vs severe) as response in dogs with CIRD. Models are ranked based on second order Akaike’s information criteria (AICc).**

| Model^*^ | Model Terms | df | logLik | AICc | delta AICc | weight |
| --- | --- | --- | --- | --- | --- | --- |
| m3 | Age (years)+ Sex (male) | 3 | -59.533 | 125.342 | 0.000 | 0.178 |
| m20 | Age (years)+ *M. cynos* | 3 | -59.580 | 125.432 | 0.090 | 0.170 |
| m4 | Age | 2 | -60.671 | 125.476 | 0.134 | 0.167 |
| m19 | Age + *M. cynos* + CPIV | 4 | -58.792 | 126.044 | 0.702 | 0.125 |
| m11 | Age + CPIV-*M. cynos* | 3 | -60.409 | 127.091 | 1.749 | 0.074 |
| m2 | Age + Sex + Number Infections | 4 | -59.427 | 127.320 | 1.978 | 0.066 |
| m13 | Age + CPIV-*M.cynos* + Age: CPIV-*M. cynos* | 4 | -59.571 | 127.601 | 2.259 | 0.058 |
| m18 | Age + *M. canis* + *M. cynos* + CPIV | 5 | -58.734 | 128.166 | 2.824 | 0.043 |
| m16 | Age + Number Infections + Age: Number Infections | 4 | -60.083 | 128.626 | 3.284 | 0.035 |
| m10 | Age + *B. bronchiseptica*-*M. cynos* + CPIV-*M. cynos* | 4 | -60.338 | 129.136 | 3.794 | 0.027 |

* Sample size for all models was n=95, which corresponded to the subset of animals studied with complete data on co-infections and molecular diagnostics. *M. cynos*= *Mycoplasma cynos*, CPIV= canine parainfluenza virus, *M. canis*= *Mycoplasma canis, B. bronchiseptica= Bordetella bronchiseptica*
